# Performance and environmental assessment of concrete made with treated industrial wastewater

**DOI:** 10.1038/s41598-026-50807-5

**Published:** 2026-05-13

**Authors:** Samuel Mohsen, Ayman Shamseldein, Engy hany Wadie, Hany Elshafie

**Affiliations:** Structural Engineering Department, Faculty of Engineering, Ain Shams University, Cairo, Egypt

**Keywords:** Recycling, Concrete, Treated industrial water, Leaching, Durability, Mechanical properties, Engineering, Environmental sciences, Materials science

## Abstract

Concrete production consumes significant quantities of potable water. This demand places increasing pressure on freshwater resources, particularly in water-scarce regions. This study investigates the feasibility of using treated industrial wastewater (TIWW) as an alternative to potable water in concrete and mortar production, while also assessing potential environmental and health risks associated with contaminant leaching. Two real TIWW sources were examined, collected from textile and food industries, alongside laboratory-prepared synthetic wastewater containing controlled concentrations of copper and zinc to isolate heavy metal effects. The experimental program was conducted in three phases. Phase I involved chemical characterization of the wastewater and comparison with code-based limits of ASTM, BS & Egyptian code of practice. Phase II evaluated the influence of TIWW on fresh, mechanical, and durability-related properties of concrete, including workability, compressive, flexural, and splitting tensile strengths, sorptivity, rapid chloride penetration, and hydration products using X-ray diffraction (XRD). Phase III focused on cement paste and mortar properties, including water consistency, setting time, compressive strength, and accelerated leaching behavior. Results indicate that using TIWW led to slight reductions in compressive strength (≤ 10%) and minor decreases in tensile and flexural strengths (< 5%), while workability was largely unaffected. Sorpitivity decreased when TIWW was used, attributed to pore-blocking effects of organic matter, whereas rapid chloride penetration increased due to higher ionic conductivity within the pore solution. Setting time retardation was limited for real TIWW but became significant when heavy metal concentrations exceeded 0.5 g/L. Accelerated leaching tests demonstrated minimal heavy metal release, confirming effective immobilization within the cementitious matrix.In conclusion, the findings confirm that TIWW can be safely used in structural concrete under controlled conditions.

## Introduction

Concrete is an essential material of infrastructure and is among the most widely used construction materials globally^1–4^. However, concrete production requires large amounts of potable water for both mixing and washing. It is estimated that the industry consumes nearly 16% of the world’s freshwater resources. At the same time, many regions are already facing severe water scarcity, which negatively impacts agriculture and food production. Therefore, reducing the use of fresh water in concrete can free up valuable resources for planting new crops or other essential and beneficial purposes^5–7^. This can be achieved by exploring alternative sources, such as treated industrial wastewater (TIWW). This research directly contributes to several United Nations Sustainable Development Goals (SDGs). By exploring the replacement of potable water with treated industrial wastewater in concrete production, it supports SDG 6 (Clean Water and Sanitation) through improved water-use efficiency and conservation of freshwater resources. It also aligns with SDG 9 (Industry, Innovation, and Infrastructure) by promoting sustainable and innovative construction practices that reduce reliance on scarce natural resources. In addition, the study addresses SDG 11 (Sustainable Cities and Communities) and SDG 12 (Responsible Consumption and Production) by encouraging the reuse of industrial wastewater, minimizing environmental impacts, and advancing eco-friendly infrastructure. Indirectly, the work contributes to SDG 13 (Climate Action) by enhancing resilience in regions facing water scarcity. Previous research studied the effect of TIWW on setting time of concrete.

Studies on the use of treated industrial wastewater (TIWW) in concrete and cement paste indicate varying effects on fresh and hardened properties, depending on the source and composition of the water and following is a summary:

1. *Setting time* is generally affected by the presence of heavy metals and organic materials.
  Babu et al.^8^ observed slight retardation in initial and final setting times with TIWW, mainly attributed to heavy metals, though the delay was minimal (5–15 min) and within ASTM and BS limits^1,2^. Similar delays were reported by Gholamreza et al.^9^(≈ 17 min).
  Haezah et al.^10^ noted a slight decrease in setting time for industrial and domestic wastewater, but a significant increase from palm oil mill wastewater due to organic content.
2. *Workability* is often reduced with TIWW with some exceptions. Gholamreza et al.^9^ reported a slump reduction of 12.5%, while Haezah et al.^10^ observed decreases of 25–50 mm, attributed to solid particles absorbing mix water. Nasseralshariati et al.^11^. confirmed that increasing TIWW content reduces workability, and Ali et al.^12^ noted variations depending on water source, with some high-salt waters slightly increasing slump.
3. *Compressive strength* was studied and found moderately affected by the TIWW used.
  Gholamreza et al.^9^ found reductions of 7.9–8.4% across mixes with different cement contents, remaining within the 10% limit allowed by BS EN 1008. Khalifa et al.^13^ reported decreasing strength with increasing wastewater content, except for a 50% mix ratio that matched control samples. Also, Babu et al.^8^ found delayed strength gain up to 180 days, after which strength matched control using TIWW from paint industry.
  Treatment level also influences strength: secondary and tertiary treated wastewater show smaller reductions, while lower treatment levels reduce compressive strength due to weak ettringite formation^14^.
  Alshariati et al.^11^ highlighted the importance of using the same water type for mixing and curing, which slightly improved strength (0.5–3.6%). Some wastewater types, such as textile or fertilizer factory effluents, even increased early compressive strength due to reactive ions^15^.
4. *Tensile and flexural strength* exhibit similar trends. Tensile strength is generally more sensitive to TIWW than compressive strength due to weaker interfacial transition zones^11^.
  Barbar Ali et al.^12^ reported reductions ranging 5–25% for treated wastewater, while untreated wastewater caused up to 70% loss depending on organic content.
  However, some sources, such as tertiary-treated domestic sewage or textile wastewater, led to increases in tensile and flexural strength^15^. Heavy metals in TIWW were found to slightly enhance flexural strength after 90 days due to positive interactions with the cement matrix^15^.
5. *Water Absorption* was found dependent on the type of water used.
  Raza et al.^16^, where they investigated the effect of different industrial wastewaters from textile factory wastewater (TFW), fertilizer factory wastewater (FFW), domestic sewerage wastewater (DSW), and service station wastewater (SSW) on the water absorption of concrete. Their study showed that the impact depends on the type of wastewater: TFW resulted in lower water absorption than potable water, while DSW caused higher absorption due to increased porosity from organic matter content.
  Similarly, Ali et al.^12^ reviewed multiple studies and noted that wastewater with minimal impurities or organic matter has little effect on concrete absorption, whereas wastewater with high organic content, such as domestic sewage, significantly increases porosity and absorption.
6. *Chloride penetration* was studied Raza et al.^16^ found that all types of wastewaters increased the chloride penetration depth at 28 days, with TFW showing a 17% increase and FFW a 29% increase. Ali et al.^12^ reported that increasing the proportion of untreated wastewater in concrete mixes could raise chloride penetration by up to 33%, attributed to reduced formation of hydration products. Conversely, using treated wastewater improved chloride penetration resistance by approximately 13% compared to untreated wastewater.

Table 1 shows a summary of the results of the literature review.

**Table 1:** Literature review summary.

Reference	Conclusion	Author
^7^	Both initial and final setting times of mortar were affected by the different types of mixing waters. As the metal concentration increased, setting times were retarded but not significantly. Using TIWW results in delay compressive strength gain up to 180 days.	G. Reddy Babu
^8^	The use of treated industrial wastewater in the cement paste affected worability by delaying both initial and final setting time, as well as reducing slump. the use of treated industrial wastewater in concrete production reduced the compressive strength of cement mortar by 7.9–8.4%	Gholamreza Asadollahfardi
^9^	Using TIWW results in workability decrease evident in a slump decrease of 25–50 mm. Some types of water results in retardation of setting time of cemente pasete, however other types such as palm oil water results In increasing setting time.	Ainul Haezah Noruzman
^10^	Replacing potable water with TIWW results in rexucing workability. Using same type ofTIWW for both mixing and curing results in slightly improved strength (0.5–3.6%).	Ehsan Nasseralshariati
^11^	Chnaging the type of TIWW have different effects on workability based on the source of water. Using untreated wastewater rsults in reduction of up to 70% of tensile and flexural strength, while using TIWW results in 5–25% reduction based on type of water. Using water with high content of organic materials results in highr effects on conrete water absorption. Increasing the proportion of untreated wastewater in concrete mixes could raise chloride penetration by up to 33%, attributed to reduced formation of hydration products.	Babar Ali
^12^	Increasing percentage of TIWW results in slight decrease in compressive strength. All concrete mixtures with wastewater replacement showed similar water absorption rates to the control mixture.	Khalifa S. Al-Jabri
^13^	Treatment level of wastewater affects strength reduction and workability, where secondary and tertiary treated wastewater shows less strength reduction and better workability results.	Khushboo Meena
^14^	Using TTWW achieves strength better than fresh water by (4.9%, 8.8%, and 6.7%) at 28 days. Heavy metals in TIWW were found to slightly enhance flexural strength after 90 days due to positive interactions with the cement matrix.	Mohamed A. E. Halawa
^15^	all types of wastewaters increased the chloride penetration depth at 28 days, with TFW showing a 17% increase and FFW a 29% increase. Results of water absorption of concrete dpends mainly on the type of water used, where some types such as (TFW) shows less waterabsorption and some types as (DSW) shows higher absorption levels.	Ali Raza

From the previous studies, it is clear that no confirmation to date on the effect of TIWW on concrete properties. Furthermore, the effect of TIWW depends on its source. Therefore, this research was initiated to study different sources of treated industrial wastewater (TIWW) and to investigate its effects on both short- and long-term concrete properties. Additionally, few studies have addressed the potential leaching of TIWW and its associated health hazards when contaminant levels are high.

**Fig. 1:**
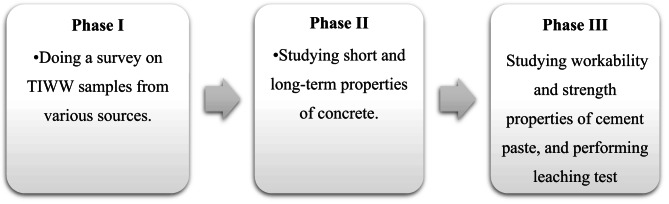
Experimental program phases.

The experimental program of this study as shown in Fig. 1 was structured into three main phases to systematically evaluate the effects of treated industrial wastewater (TIWW) on concrete and cement mortar. Phase I involved a comprehensive survey of TIWW from multiple industrial sources, aiming to identify the chemical constituents present and establish baseline data for evaluating their potential impact. Phase II focused on investigating the influence of TIWW from two selected factories on short- and long-term concrete properties, including workability (slump and slump loss), strength characteristics (compressive, flexural, splitting tensile strengths, and modulus of elasticity), and durability-related properties such as Sorpitivity and rapid chloride penetration. Phase III examined the effects of different water types, including TIWW and laboratory-prepared heavy metal solutions, on the workability and strength of cement paste, specifically addressing water consistency, initial and final setting times, and compressive strength. Finally, leaching tests were carried out to assess potential health hazards associated with the use of TIWW, providing essential data on environmental and safety considerations. Collectively, these phases offer a comprehensive framework for understanding how various compositions of industrial wastewater, including potential contaminants, influence the essential properties of concrete and cement mortar. Research gap has been noticed to be insufficient industrial wastewater sources studied globally, as well as insufficient environmental impact studies conducted.

## Experimental program – materials and methodology

### Research plan

The experimental program of this study was organized into three sequential phases to systematically investigate the effects of treated industrial wastewater (TIWW) on concrete and cement mortar.

#### Phase I: Chemical analysis of water samples

The first phase focused on collecting industrial treated water samples from major industrial sectors and conducting chemical analyses to provide an initial assessment of their potential impacts by comparing with codes limits.

#### Phase II: Concrete samples

In this phase the effect of collected water samples on concrete specimens were investigated. the carried-out tests included workability (slump and slump loss), mechanical properties (compressive, flexural, splitting tensile strengths, and modulus of elasticity), as well as durability-related characteristics, such as Sorpitivity and rapid chloride penetration.

#### Phase III: Cement mortar and paste samples

In this phase, the effects of the collected TIWW on cement paste and mortar samples were investigated. In addition, to isolate the influence of individual heavy metals, synthetic industrial wastewater with varying concentrations was prepared and tested. The experimental program included water consistency, initial and final setting times, and compressive strength measurements. At the end, leaching tests were carried out to evaluate potential environmental and health hazards associated with TIWW. This phase provided critical data regarding the safety and sustainability implications of using treated industrial wastewater in construction materials.

### Materials

#### Mixing water

This study utilized four main types of water to investigate their effects on the properties of concrete, cement paste, and cement mortar. Potable water (PW) was used as a control to establish baseline performance. Two treated industrial wastewater (TIWW) was collected directly from two industrial facilities which are food production and textile manufacturing to assess the real-world impact of wastewater containing multiple chemical constituents. Finally, synthetic heavy metal water was prepared in the laboratory with controlled concentrations of selected metals, enabling the isolated evaluation of heavy metal effects on cementitious materials. The types of TIWW and synthetic water was discussed in detail in the following sections.

##### Treated industrial wastewater from textile industry (TETI)

A water sample was collected from the industry after the treatment process. The treatment process consists of four main stages:

1. Flocculation: Coagulants form micro flocs that bind suspended solids, oils, and contaminants.
2. Lamella Settling Tank: Gravity settles the denser particles, leaving clarified water at the top.
3. Physical Treatment: Pressurized air removes light impurities, such as oils, via flotation and skimming.
4. pH Neutralization: Hydrochloric acid adjusts water pH to a neutral range (7.5–8.5) before discharge.

##### Treated industrial wastewater from food industry (TEFI)

A water sample was collected from food industry after treatment process. The factory operates a multi-stage wastewater treatment system:

1. Aeration: Oxygen promotes aerobic microbial breakdown of organic matter.
2. Chemical Addition: Slaked lime and cationic polymers adjust pH and promote flocculation.
3. Lamella Tank: Settles suspended solids for clarified water separation.
4. Biological Treatment: Moving Bed Biofilm Reactor (MBBR) reduces COD and BOD.
5. Sand and Carbon Filtration: Removes remaining impurities for safe discharge.

##### Synthetic heavy metal water

To isolate the effect of heavy metals on concrete and mortar, synthetic water containing Copper (Cu) and Zinc (Zn) was prepared in the laboratory. Direct dissolution of metal powders in water was ineffective, so metals were first dissolved in diluted acid and then neutralized with an alkaline solution to maintain a neutral pH of 7. Two concentrations were prepared for each metal:

- Copper: 0.5 and 1.0 g/L.
- Zinc: 1.0 and 2.0 g/L.

These controlled solutions allowed the study of the specific effects of heavy metals on cementitious material properties, independently of other wastewater constituents.

#### Aggregates

For concrete specimens, the coarse aggregate consisted of crushed dolomite with specific gravity of 2.7 and the fine aggregate was natural fine sand with specific gravity of 2.5. For mortar specimens, only fine aggregate consisting of natural sand with a specific gravity of 2.5 was used. All the properties of aggregates are conforming to ASTM C33^17^. Tables 2 and 3 shows the sieve analysis curves of the fine and coarse aggregate used.

**Table 2:** Sieve analysis results of coarse aggregate.

Sieve No.(mm)	37.5	31.5	28	20	10	5
Passing Percentage (%)	100	100	100	100	31.2	5.2

**Table 3:** Sieve analysis results of fine aggregate.

Sieve No.(mm)	4.75	2.36	1.18	0.6	0.3	0.15
Passing Percentage (%)	98.0	90.1	80.3	39.4	11.2	5.0

#### Cement

The cement used in all concrete and mortar mixes was Ordinary Portland Cement (CEM I 42.5 N), conforming to the requirements of EN 197^18^. Table 4 shows the physical properties of cement used.

**Table 4:** Physical properties of cement.

Parameter	Min. 2 days compressive strength (*N*/mm^2^)	Min. 28 days compressive strength (*N*/mm^2^)	Initial Setting time (min.)	Soundness (mm)
Result	10.0	42.5	≥ 60	1

#### Mix design for concrete samples

The mix design was carried out based on the British standards guidelines. No admixtures were used in the mix. Detailed information on the specific mix proportions used can be found in Table 5, which outlines the exact quantities of each material used in the concrete formulation.

**Table 5:** Mix proportions needed to produce 1 m^3^ of concrete.

	Cement	Free water	Fine Aggregates	Coarse Aggregates
Weight (Kg)	340	190	605	1225

As for the mortar mix, ASTM C-109^19^ was followed, using 500 gm of cement, 1375 gm of sand and 242 ml of water to produce six mortar cubes with dimensions of 5 × 5 × 5 cm.

#### Mixing procedure

For concrete samples the following procedures are followed, coarse aggregate was properly washed to make sure no fine materials or impurities are present prior to mixing and was left to dry to be in the saturated dry surface condition. Then, quantities needed of cement, fine aggregate, and coarse aggregate were weighted and then dry-mixed in a pan mixer for one minute, after which approximately 70% of the mixing water was added gradually while mixing continued for two minutes. The remaining water was then added and mixing proceeded for an additional two minutes until homogeneous concrete was obtained. The fresh concrete was placed into pre-oiled molds in two layers, each compacted using a vibrating table, and the surface was leveled before curing for 24 h and subsequent water immersion until testing.

Cement mortar was mixed using a standard mechanical mixer by first combining cement and fine aggregate at low speed for 30 s, followed by gradual addition of water over the next 30 s, then mixing at medium speed until reaching proper consistency. The fresh mortar was cast into molds in two layers with proper compaction, covered for 24 h, demolded, and then cured in water until the designated testing ages.

### Specimen preparation and testing set up

For phase I, only chemical test was carried out for water samples. For Phase II, tested carried out included compressive strength, flexural strength, splitting tensile strength, slump. Along with long-term properties of concrete including Sorpitivity and rapid chloride penetration and finally X ray diffraction test (XRD). Phase III focused on the cement paste and cement mortar properties, including water consistency, initial and final setting time for cement paste, and compressive strength of cement mortar. at the end of phase III, leaching tests were carried out according to ASTM C1308^20^.Table 6 shows all the carried-out tests with used standards .

**Table 6:** Tests.

Series	Phase	Test	Standard
1	I	Chemical analysis test	ASTM C1603^1^
2	II	Compressive strength testing for hardened concrete	BS-EN 12390^21^
3		Standard test method for splitting tensile strength of cylindrical concrete specimens	ASTM C496^22^
4		Standard test method for flexural strength of concrete (using simple beam with center- point laoding)	ASTM C293^23^
5		Standard Test Method for Measurement of Rate of Absorption of Water by Hydraulic-Cement Concretes	ASTM C1585^24^
6		Standard Test Method for Electrical Indication of Concrete’s Ability to Resist Chloride Ion Penetration	ASTM C1202^25^
7		XRD	ASTM E1621^26^
8	III	Standard test method for normal consistency of hydraulic cemente paste	ASTM C 0187^27^
9		Standard test method for time of setting of hydraulic cement by vicat needle	ASTM C0191^28^
10		Standard test method for compressive strength of hydraulic cement mortar	ASTM C109^19^
11		Slump of hydraulic cement concrete	ASTM C0143^29^
12		Standard test method for accelerated leach test for measuring contaminated releases from solidified waste	ASTM C 1308^20^

#### Chemical tests

Chemical analysis of water is a key factor to understand behavior of concrete and mortar in which this water is used. Hence, key parameters were examined, including pH value, total dissolved solids (TDS), chloride and sulphate concentrations, chemical oxygen demand (COD), biochemical oxygen demand (BOD), and the concentrations of selected heavy metals such as zinc (Zn), copper (Cu), and chromium (Cr).

#### Compressive strength for concrete specimens

**Fig. 2:**
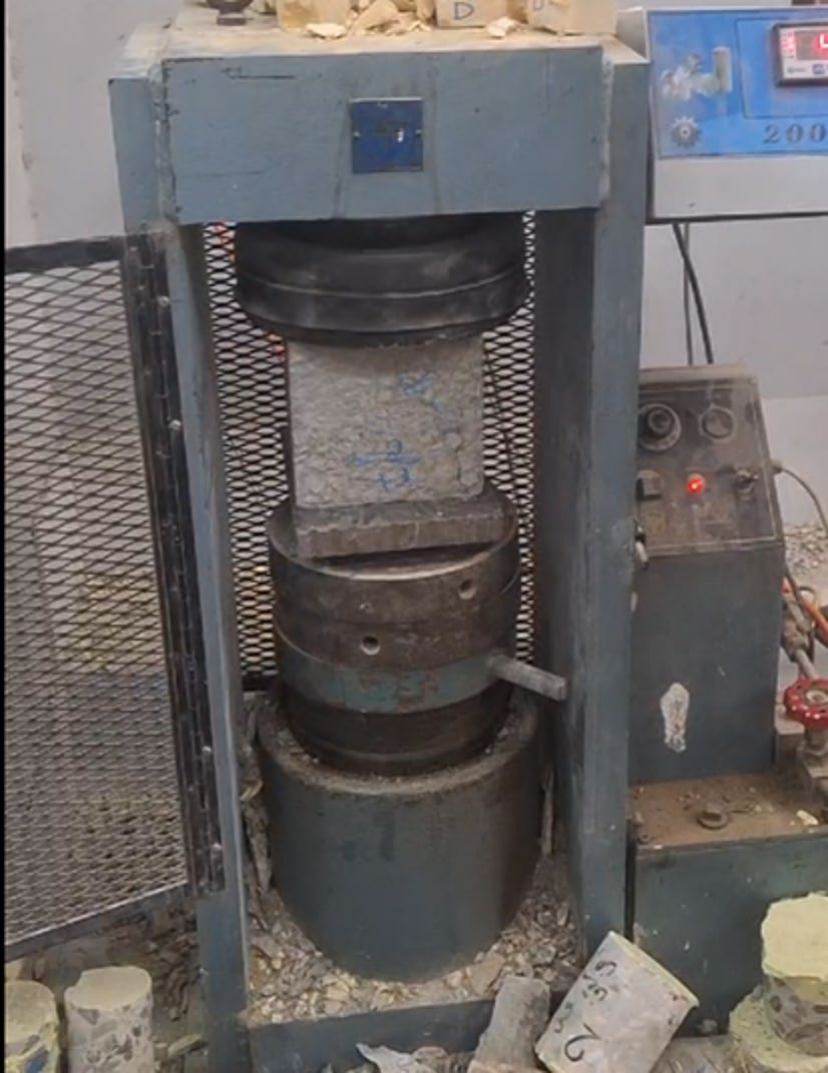
Compressive strength test.

Compressive strength tests were conducted on concrete cube specimens with dimensions of 15.8 × 15.8 × 15.8 cm, as shown in Fig. 2. Tests were performed at different curing ages (3, 7, 28, and 90 days) to capture strength development.

#### Splitting tension for concrete specimens

**Fig. 3:**
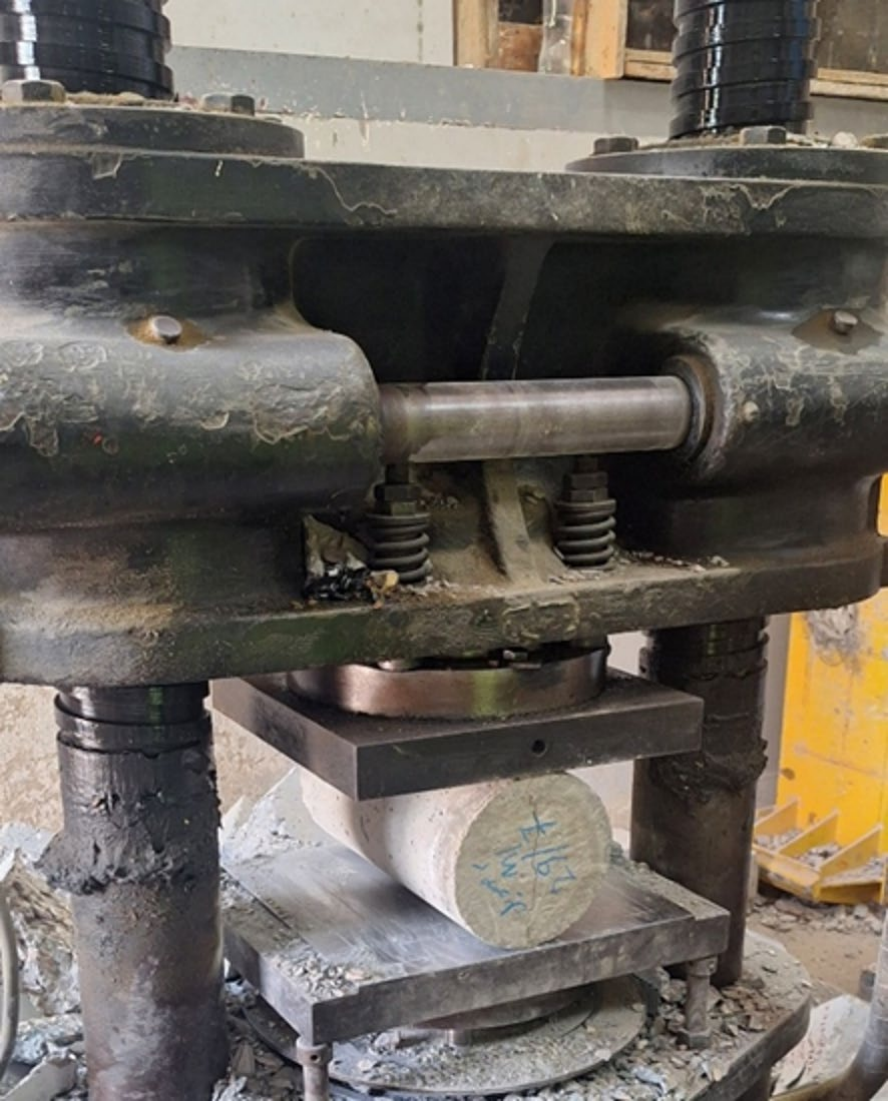
Splitting tensile test.

Cylindrical specimens with a diameter of 150 mm and a height of 300 mm were prepared and tested for splitting tensile strength at curing ages of 7 and 28 days. Figure 3 shows the conducted test.

#### Flexural strength testing for concrete

**Fig. 4:**
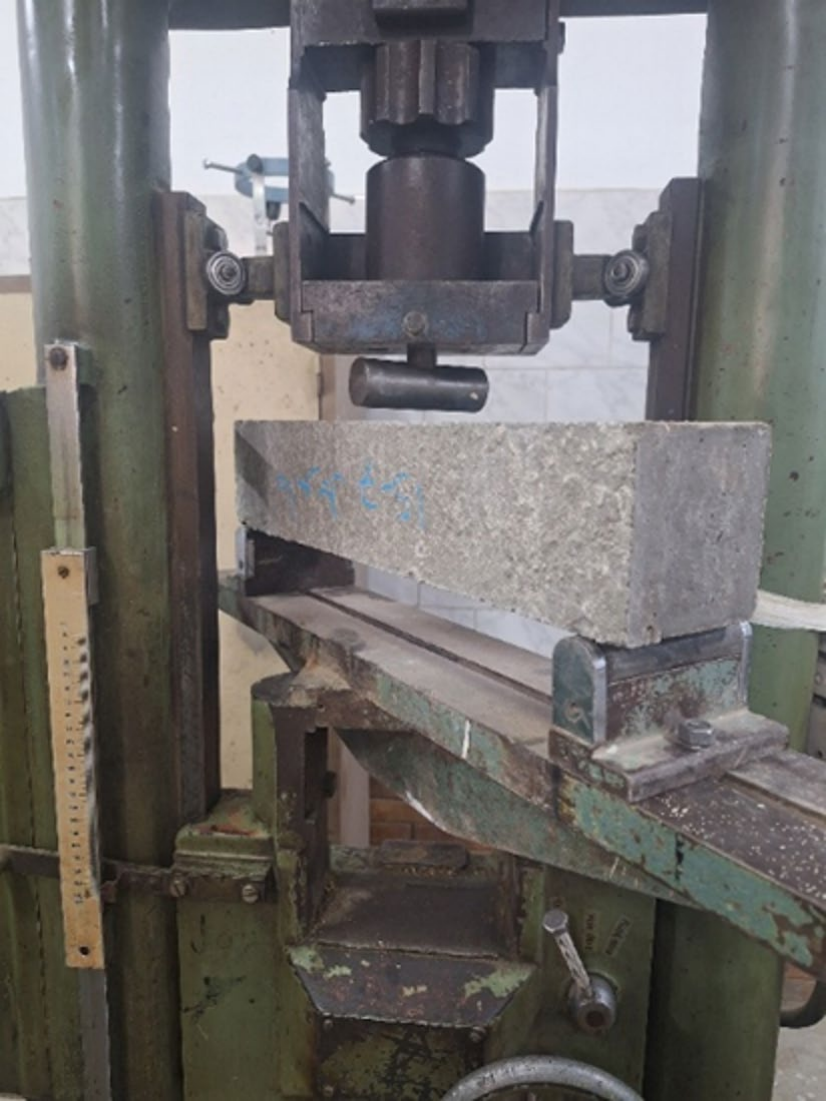
Flexural strength test.

Beams specimens with cross-sectional dimensions of 100 × 100 mm and a length of 500 mm were tested. Flexural strength tests were conducted at 28 days using a three-point loading configuration and a span of 450 mm, as illustrated in Fig. 4.

#### Sorpitivity testing for concrete

**Fig. 5:**
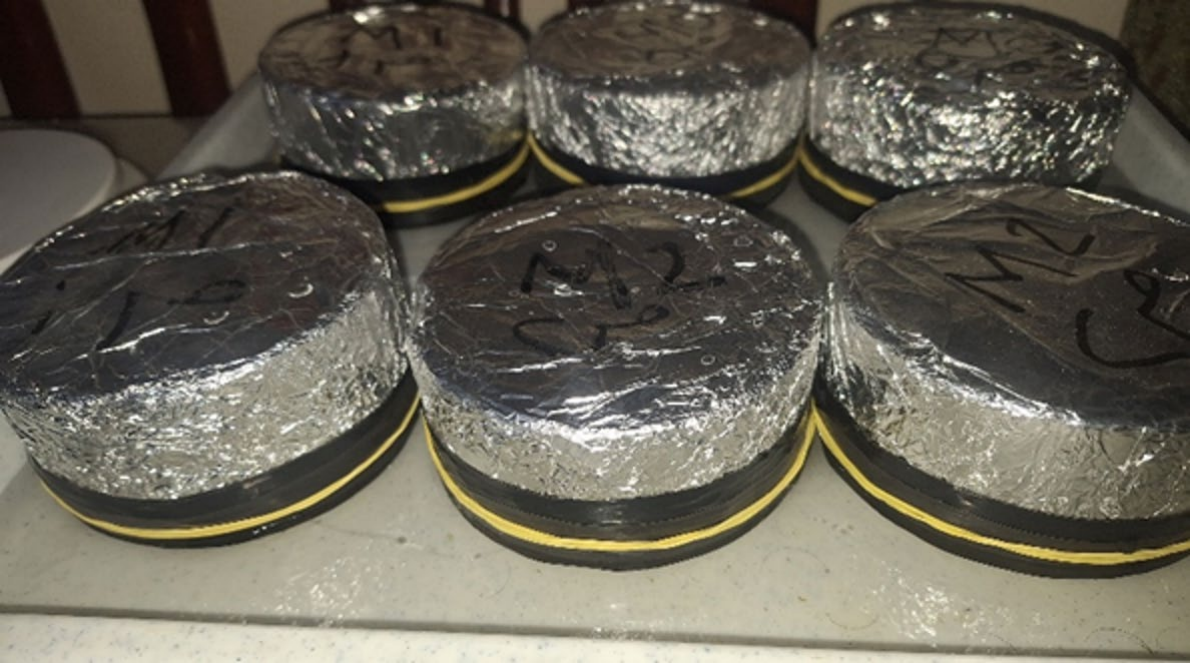
Sorpitivity test.

This test was conducted to determine the rate of water absorption by concrete, as illustrated in Fig. 5. Although the test is not directly related to the strength properties of concrete, it is considered a key durability indicator. The water absorption test is particularly crucial when concrete produced with TIWW is intended for use in practical construction applications, as it provides insight into the concrete’s pore structure and potential long-term durability. According to ASTM C1585^21^, the required specimen is a disk with a diameter of 100 mm and a height of 50 mm.

#### Rapid chloride penetration testing for concrete

**Fig. 6:**
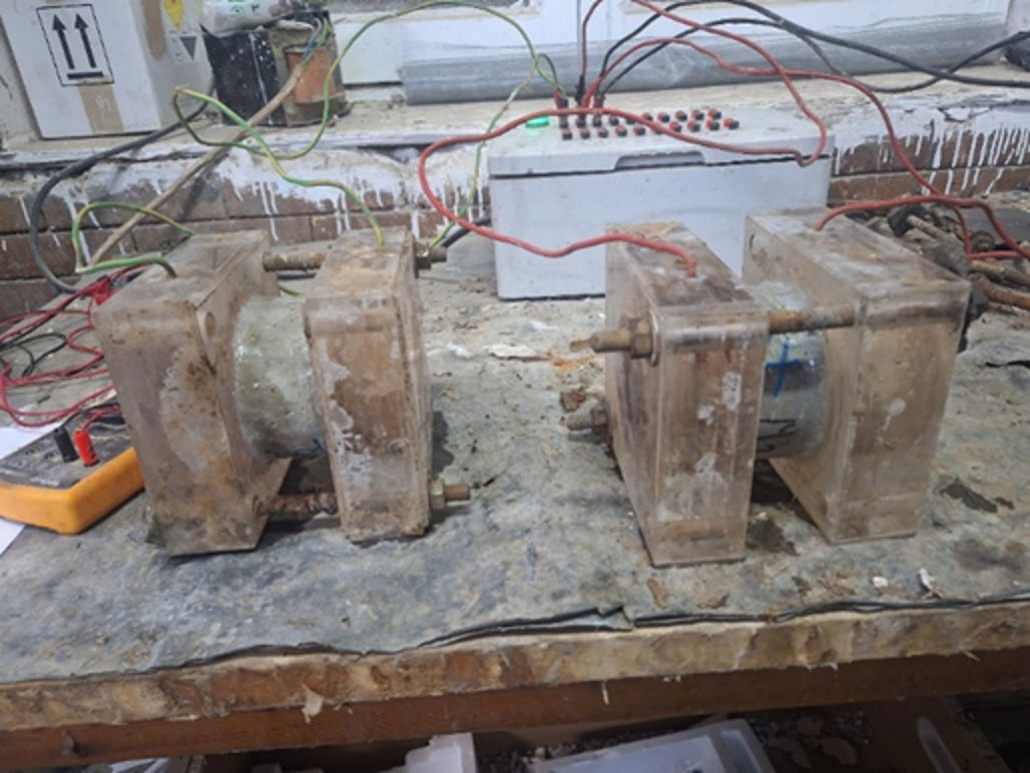
Rapid chloride penetration test.

The Rapid Chloride Permeability Test (RCPT) was conducted using cylindrical specimens with a diameter of 100 mm and a height of 50 mm, as shown in Fig. 6. This test is commonly employed to assess the resistance of concrete to chloride ion penetration, a key factor influencing the long-term durability of reinforced concrete structures, particularly in marine environments or regions exposed to de-icing salts.

#### X-ray diffraction test (XRD)

A complete X-ray diffraction (XRD) test was performed on a sample of the tested concrete specimens in order to identify the components of the specimen and, from that, determine a possible explanation for the observed behavior. The main goal of using XRD is to detect and quantify crystalline phases in cementitious materials and enable a deeper understanding of the hydration process and microstructural development.

#### Normal consistency testing for cement mortar

**Fig. 7:**
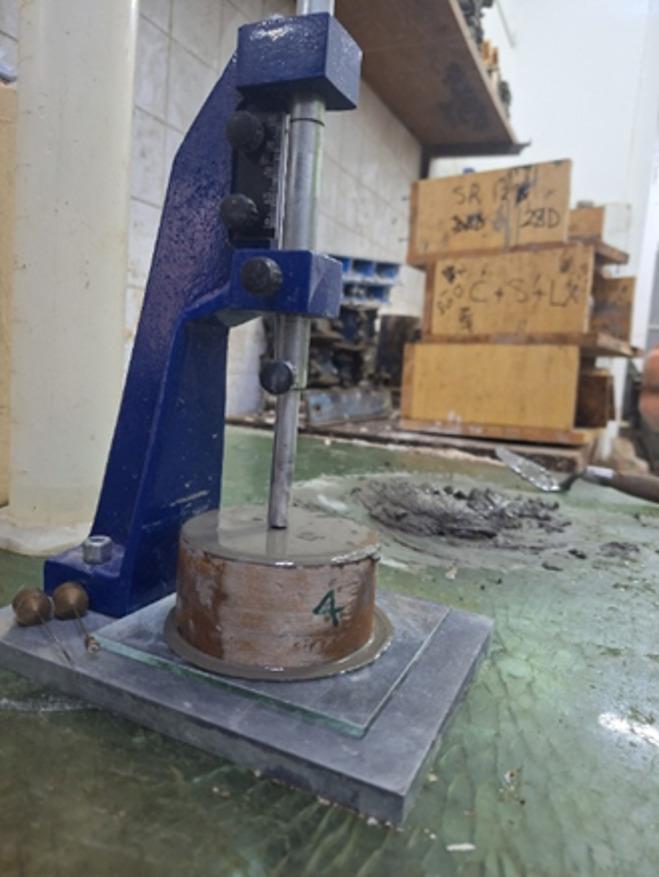
Consistency test.

This test was performed in accordance with ASTM C187^24^ to determine the amount of water required to produce a cement paste with normal consistency. The cement paste is considered to have achieved normal consistency when the penetration depth of a standard plunger reaches exactly 10 mm from the top of the specimen, as shown in Fig. 7.

#### Initial and final setting time of cement paste

**Fig. 8:**
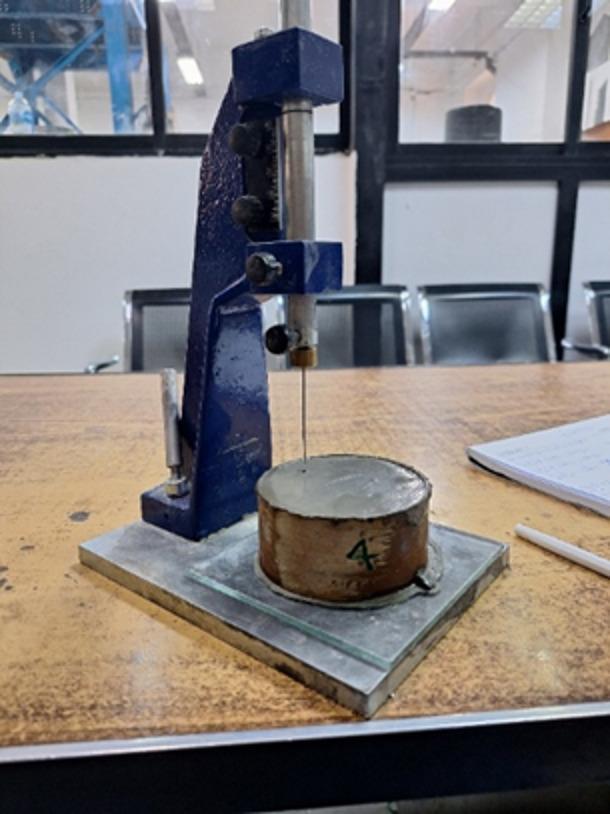
Initial setting time test.

This test was performed as shown in Fig. 8, to determine the initial and final setting times of cement paste prepared using the water content established from the water consistency test.

#### Compressive strength of cement mortar

**Fig. 9:**
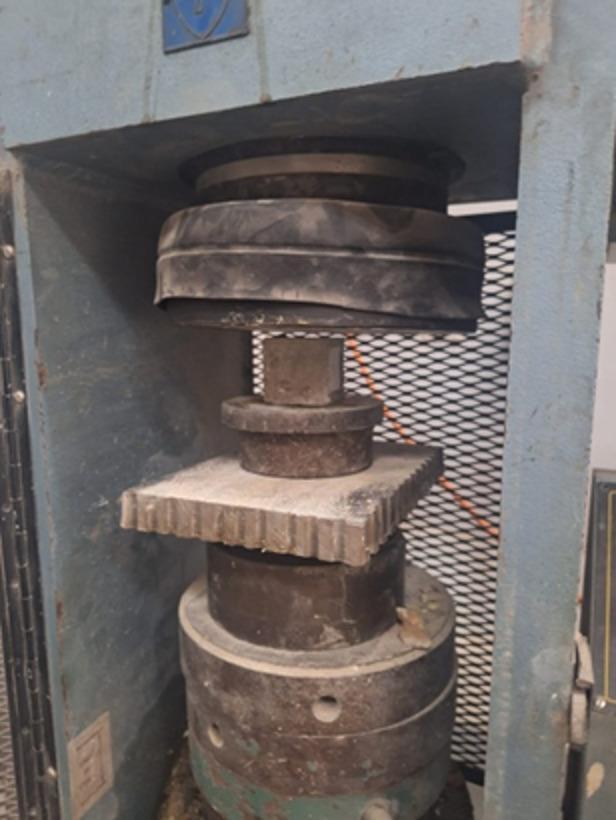
Cement mortar compressive test.

This test was conducted in accordance with ASTM C109^26^ to determine the compressive strength of mortar cubes with dimensions of 5 × 5 × 5 cm, as shown in Fig. 9. This test is particularly important because most standards specify the 7-day compressive strength of cement mortar as the acceptance criterion for the type of water used in mixing.

#### Accelerated leach test for cement paste

**Fig. 10:**
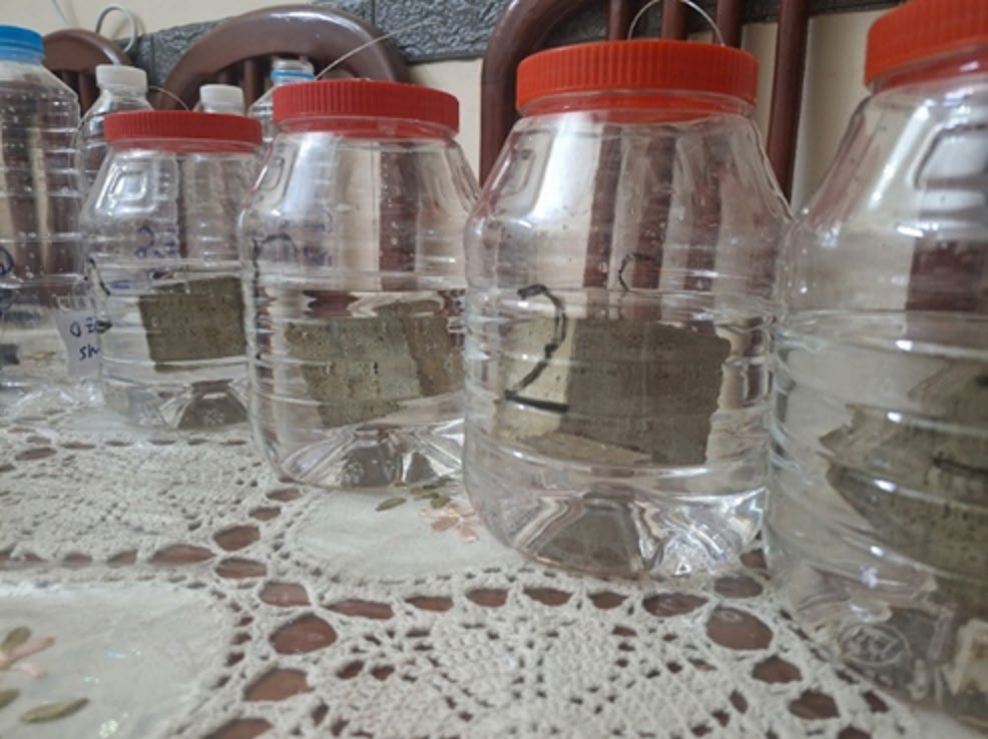
Cement mortar compressive test specimens.

Accelerated leaching test was conducted in accordance with ASTM C1308 as shown in Fig. 10 to evaluate the mobility of heavy metals - specifically zinc (Zn) - from cement mortar specimens. As Zn had the highest percentages of heavy metals in the tested treated industrial wastewater. The primary objective of this investigation is to assess whether the use of heavy metal-containing water in mortar production could lead to the release of contaminants through surface leaching, posing risks to surrounding environment.

## Test results and discussion

### Phase I

Treated wastewater samples extracted from 2 factories were chemically analyzed to determine their chemical constituents.

In this phase, the analysis focused on critical water quality parameters such as pH, total dissolved solids (TDS), chloride and sulphate content, chemical oxygen demand (COD), biochemical oxygen demand (BOD), along with concentrations of selected heavy metals including zinc (Zn), copper (Cu), and chromium (Cr). The measured values were then compared against the allowable limits outlined in Egyptian code of practice. Table 7 Shows a summary of the chemical analysis of water samples.

**Table 7:** Chemical analysis of water.

Test	Unit	Water source			Limitations of ECP
		PW	TEFI	TETI	
pH	--	7.0	8.5	7.5	> 7.0
TDS	mg/L	250	300	780	< 2000
Chlorides	mg/L	30.0	75.9	227.9	< 500
Sulphates (SO_4_)	mg/L	25.0	91.6	146.2	< 2000
Zinc (Zn)	mg/L	0	0.32	0.68	100
Copper (Cu)	mg/L	0	0.11	0.24	--
Chromium (Cr)	mg/L	0	0.023	0.094	--
BOD	mg/L	2.0	28.4	53.7	--
COD	mg/L	10.0	43.2	71.4	--

It is observed that all measured parameters were within the acceptable limits, except for COD and BOD, which exceeded the standard values typically reported for potable water. The elevated COD and BOD values can be attributed to the presence of oxygen-demanding compounds, primarily organic matter. Such compounds can interfere with the hydration process of cement, leading to delayed setting in mortar and concrete. In addition, their large surface area may adsorb onto cement particles, interfering with the hydration reactions and ultimately reducing the strength development.

### Phase II

#### Compressive strength

**Fig. 11:**
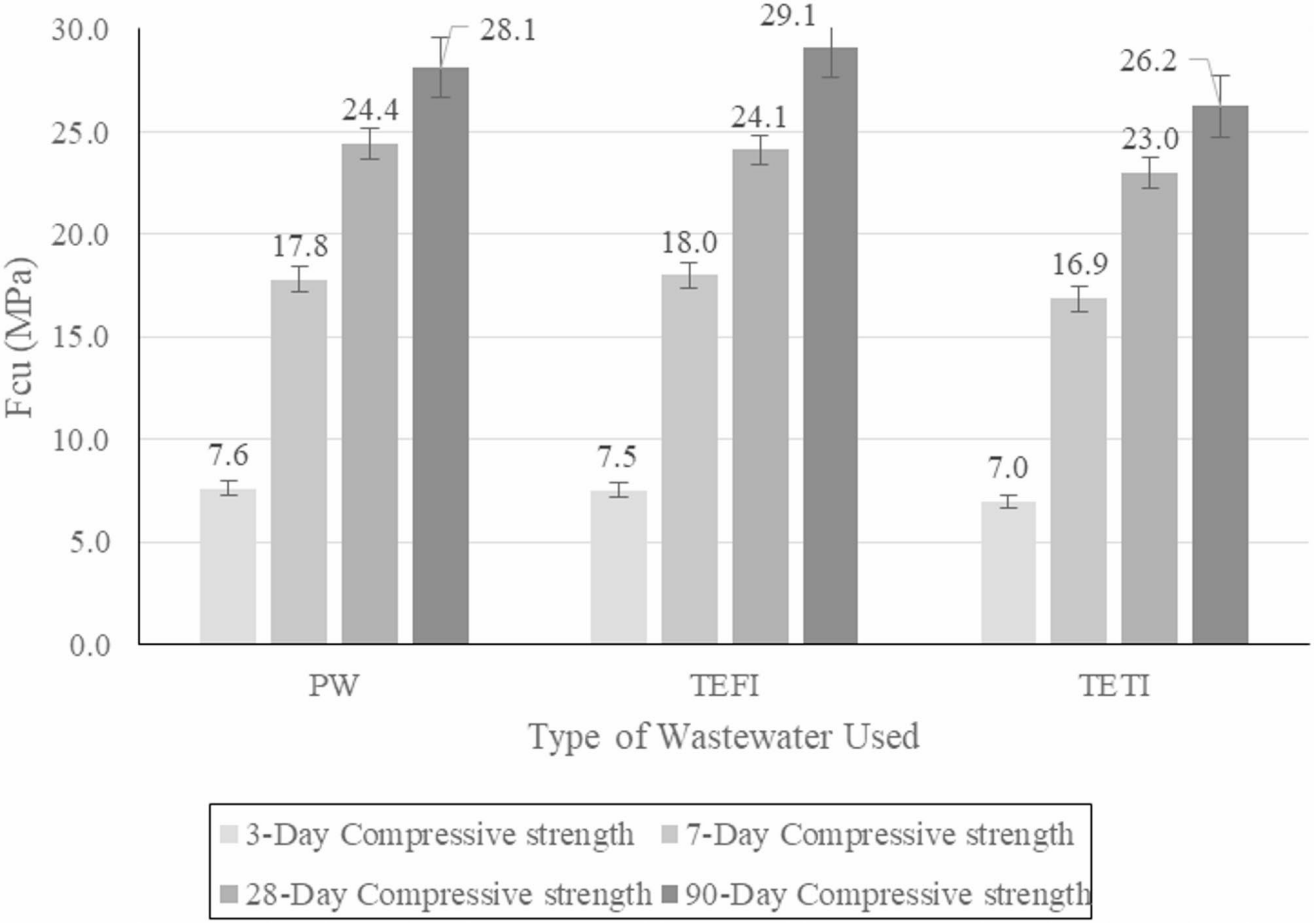
Compressive strength of concrete cubes.

Compressive strength test was performed on concrete cubes at 3, 7, 28, 90 days. The results shown in Fig. 11 show that there is a general reduction in compressive strength of concrete cubes when replacing potable water with TIWW, TEFI always has better results than TETI where at 3 days TEFI shows a reduction of 1.1% while TETI shows a reduction of 8.4%, at 7 days TEFI shows an increase of 1.1% while TETI shows a reduction of 5.3%, at 28 days TEFI shows a reduction of 1.2% while TETI shows a reduction of 5.7%. This aligns with findings by^9,13^.

Reduction in compressive strength can be linked to high amounts of oxygen demanding compounds; the decomposition of these products can disrupt the interfacial transition zone (ITZ) between aggregates and cement paste which may result in microcracking and weak zones that reduce the overall compressive strength, this aligns with the conclusion made by^11^.

#### Flexural and splitting tensile strength

Splitting tensile strength and flexural strength testing was performed on samples made with TIWW. Results shown in Table 8 show a slight reduction in both flexural and tensile strength.

Using TEFI results in a reduction of 1.2% in splitting tensile strength at 7-days and 1.1% at 28-days while using TETI results in a reduction of 2.4% in splitting tensile strength at 7-days and 2.7% at 28-days. Although TEFI shows better results than TETI. However, these reduction values are very small and insignificant; thus, it is concluded that the effect of using TWFI & TWTI on splitting tension strength is very low.

A similar conclusion was made when studying flexural strength. Using TEFI results in a reduction of 2.7% and using TETI results in a reduction of 4.6%, which again is very small and insignificant. This reduction behavior aligns with findings of^12^.

**Table 8:** Splitting tensile and flexural strength (MPa).

Type of Water used	7-Day Splitting Tensile Strength (MPa)	28-Day Splitting Tensile Strength (MPa)	28-Day Flexural Strength (MPa)
PW	1.64	1.81	5.14
TEFI	1.62	1.79	5.00
TETI	1.60	1.76	4.90

#### Slump and slump loss

**Fig. 12:**
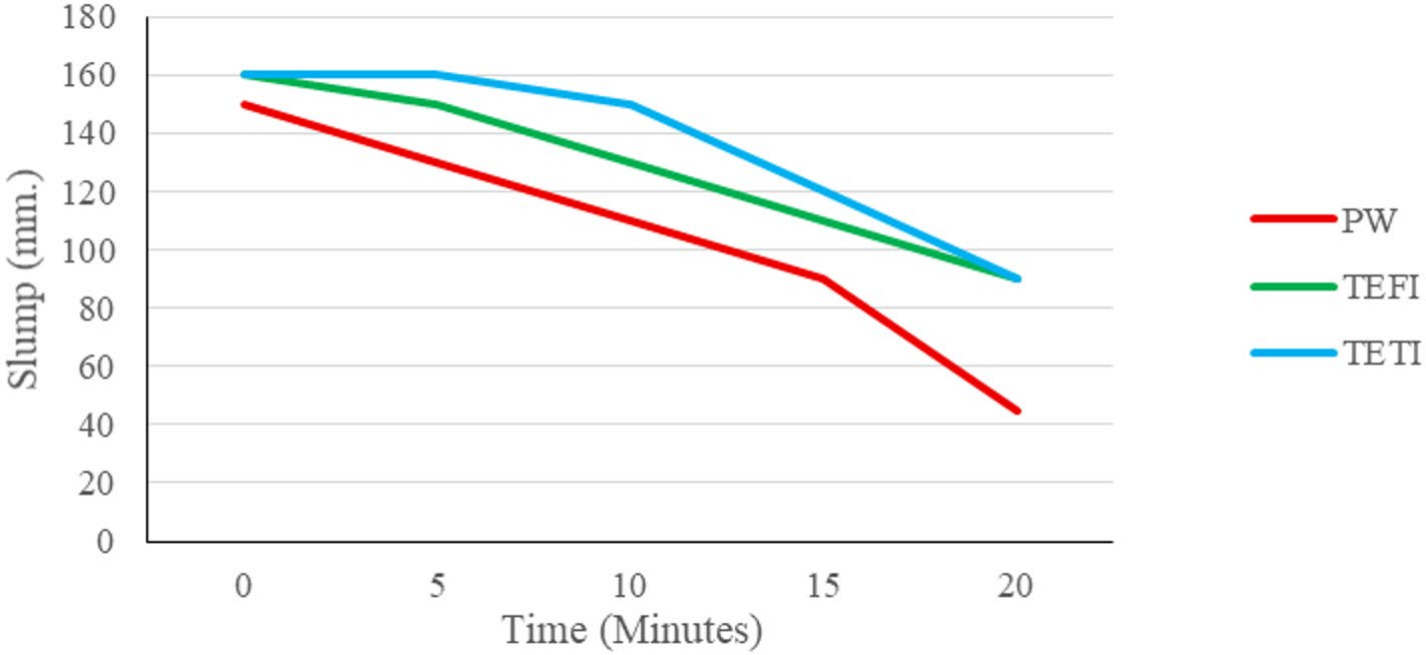
Slump loss of concrete mixes.

Standard slump test was performed for the 3 concrete mixes, and slump was recorded every 5 min for the first 20 min immediately after mixing to be able to draw the slump loss curves for the 3 mixes. Initial slump values were very close for the 3 mixes where PW mix had a slump of 150 mm, both TWFI & TWTI had a slump of 160 mm. Slump loss data shown in Fig. 12 show that for the first 10 min PW mix had less slump loss, which means better workability. This shows less slump loss and better workability results than what was concluded by^9–11^.

#### Sorpitivity

Sorpitivity test was performed and Total absorption results shows that the control mix (PW) has the highest capillary absorption (8.4 mm), TWFI mix had a total absorption of 7.45 mm and TWTI had a total absorption of 7.38 mm, this can be explained by the fact that high amounts of oxygen demanding compounds (organic matter or bacteria) can cause blocking of the capillary pores thus reducing the passage of water through this capillary pores, and this aligns with the findings of^12^ which shows water with highest organic impurities having the highest effect on pore blocking. Besides blocking the capillary pores these compounds might cause reducing the connectivity between the existing capillary pores.

**Fig. 13:**
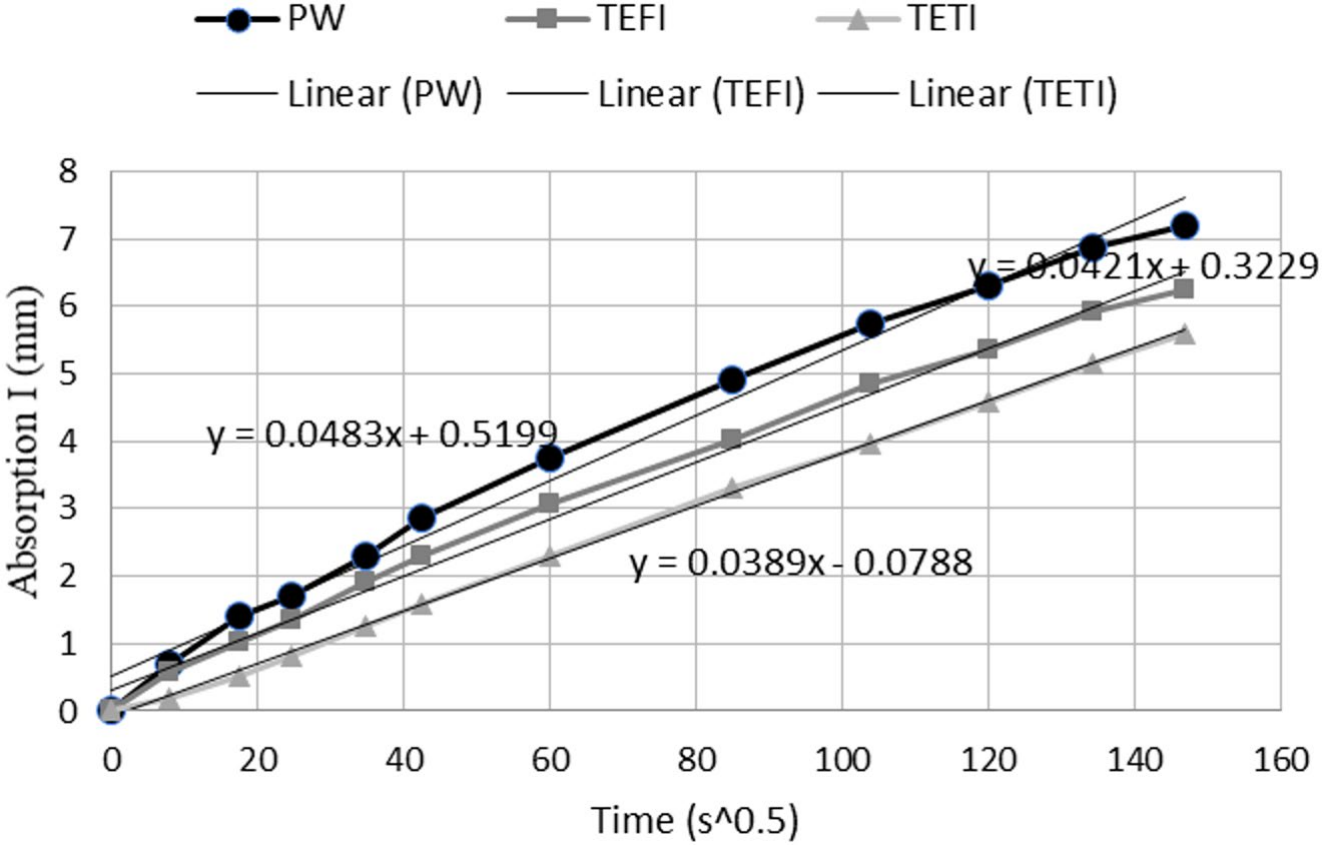
Initial rate of absorption of concrete mixes.

Initial rate and secondary rate of absorption are determined by drawing the absorption depth vs. (time)^0.5^, the initial rate is calculated as the slope of the best fitting line till a time of 6 h as shown in Fig. 13.

#### Rapid chloride penetration

Rapid chloride penetration was performed according to ASTM C1202^25^, results show that both PW and TEFI have very close quantity of charges passed (3620.97 and 3547.20 coulombs respectively) while TWTI have higher amounts of charge passed (3902.41 coulombs) which aligns with previous findings for strength properties that show worse results for TETI. This can be a little bit contradicting the water absorption through capillary action results, since TETI has the least capillary absorption. However, this can be explained by the fact that capillary absorption reflects how water is drawn into the pore system of unsaturated concrete by capillary suction. While RCPT measures total electrical charge passed, which is highly influenced by ionic conductivity — not just pore structure, and this indicates higher ionic content in pores for TWTI. This is slightly better results than what was achieved by^16^.

Also, the XRD results showed a reduction in the intensity of key hydration products in concrete prepared with treated industrial wastewater, indicating a slight modification in the hydration process and microstructural development. Such changes in hydration can influence the pore structure and hence explaining the results of Sorpitivity and RCPT.

Table 9 shows the classification of the chloride’s ion permeability according to ASTM C1202^25^, which shows that although TETI has the highest chloride Ion permeability, all 3 types of water are considered to have moderate chloride permeability.

**Table 9:** Chloride ion permeability.

Type of water used	Quantity of charges passed (Coulombs)	Chloride ion permeability
PW	3620.97	Moderate
TWFI	3547.20	Moderate
TWTI	3902.41	Moderate

#### X-ray diffraction (XRD)

**Fig. 14:**
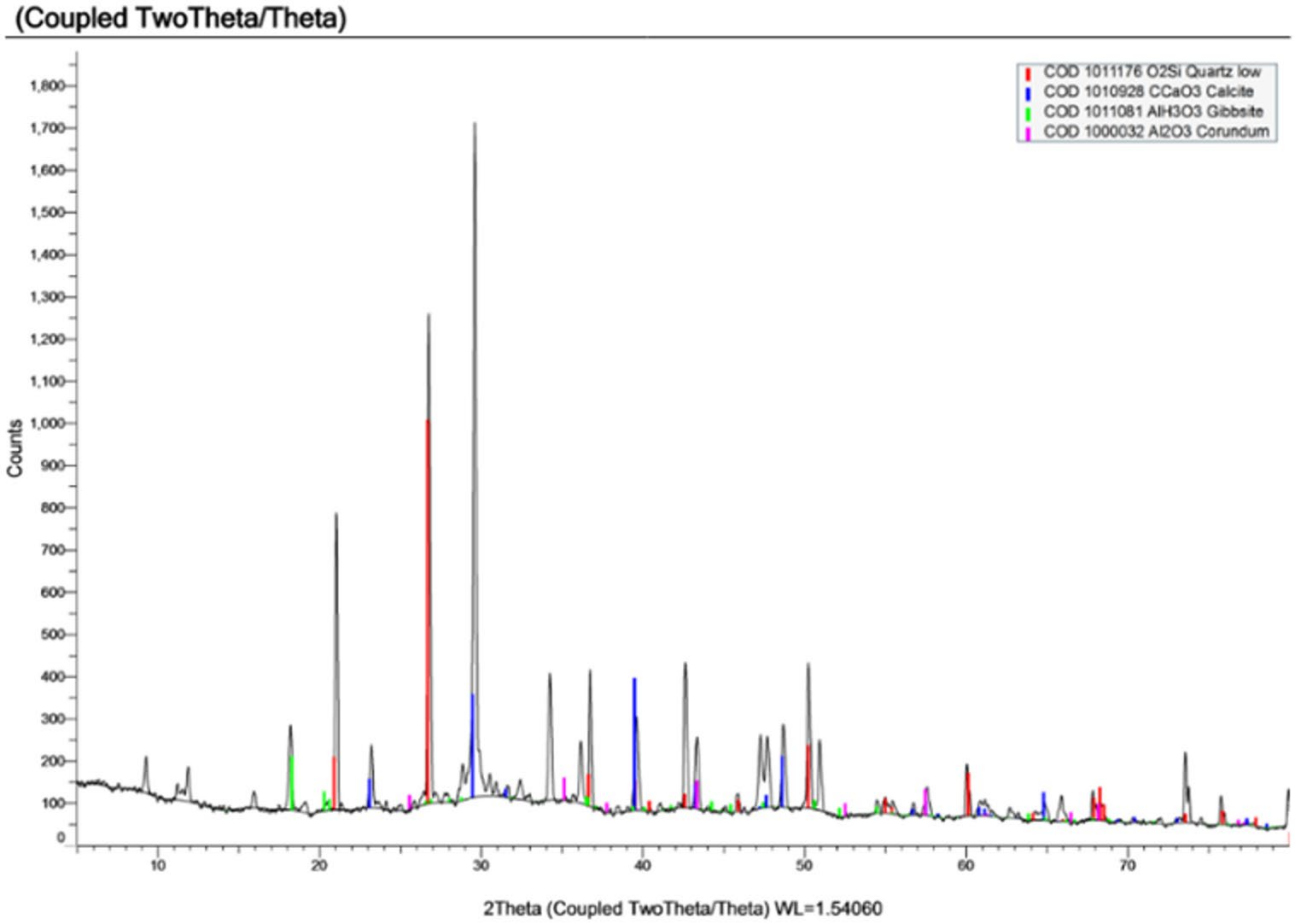
XRD of sample made with potable water.

**Fig. 15:**
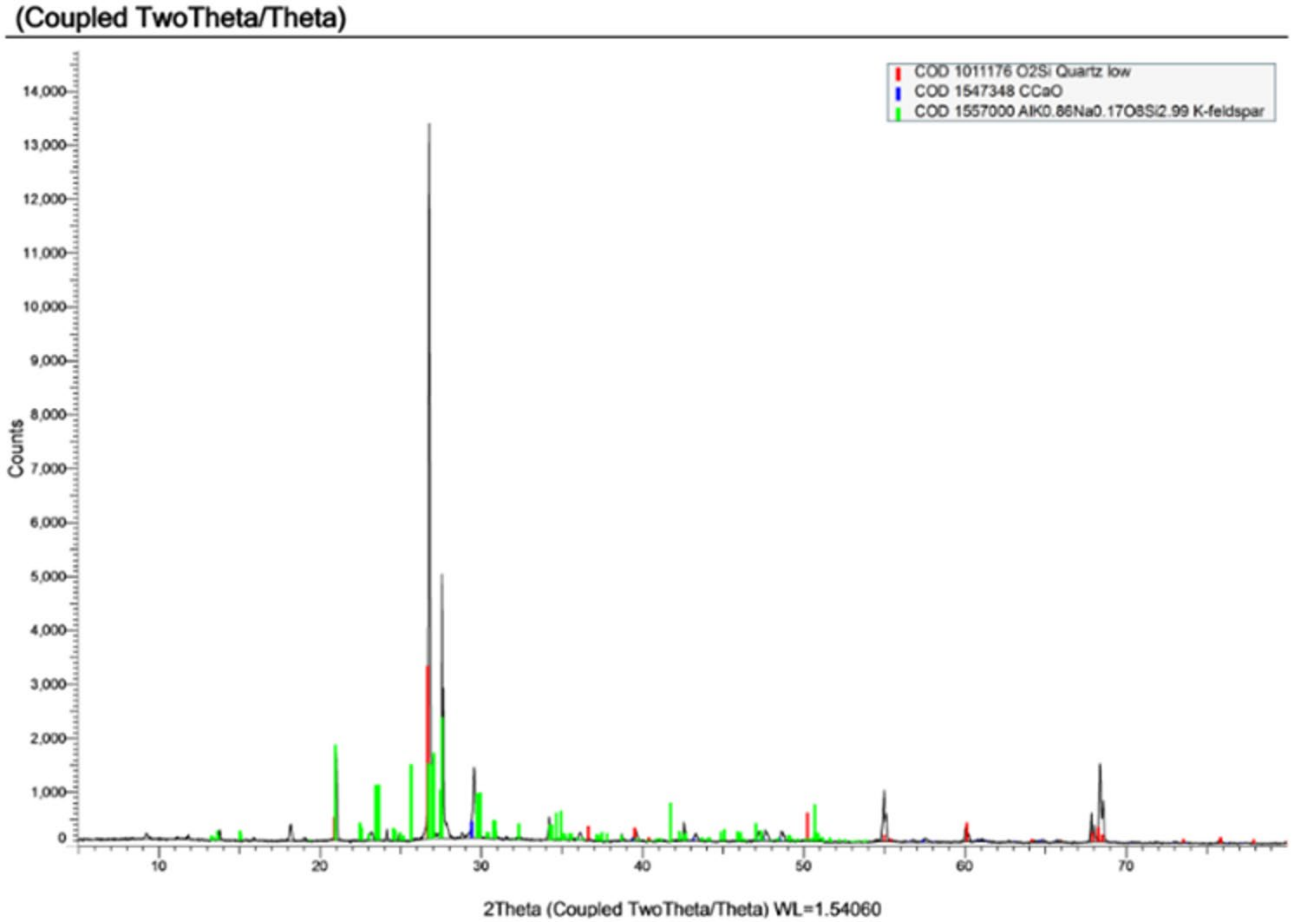
XRD of sample made with TEFI.

**Fig. 16:**
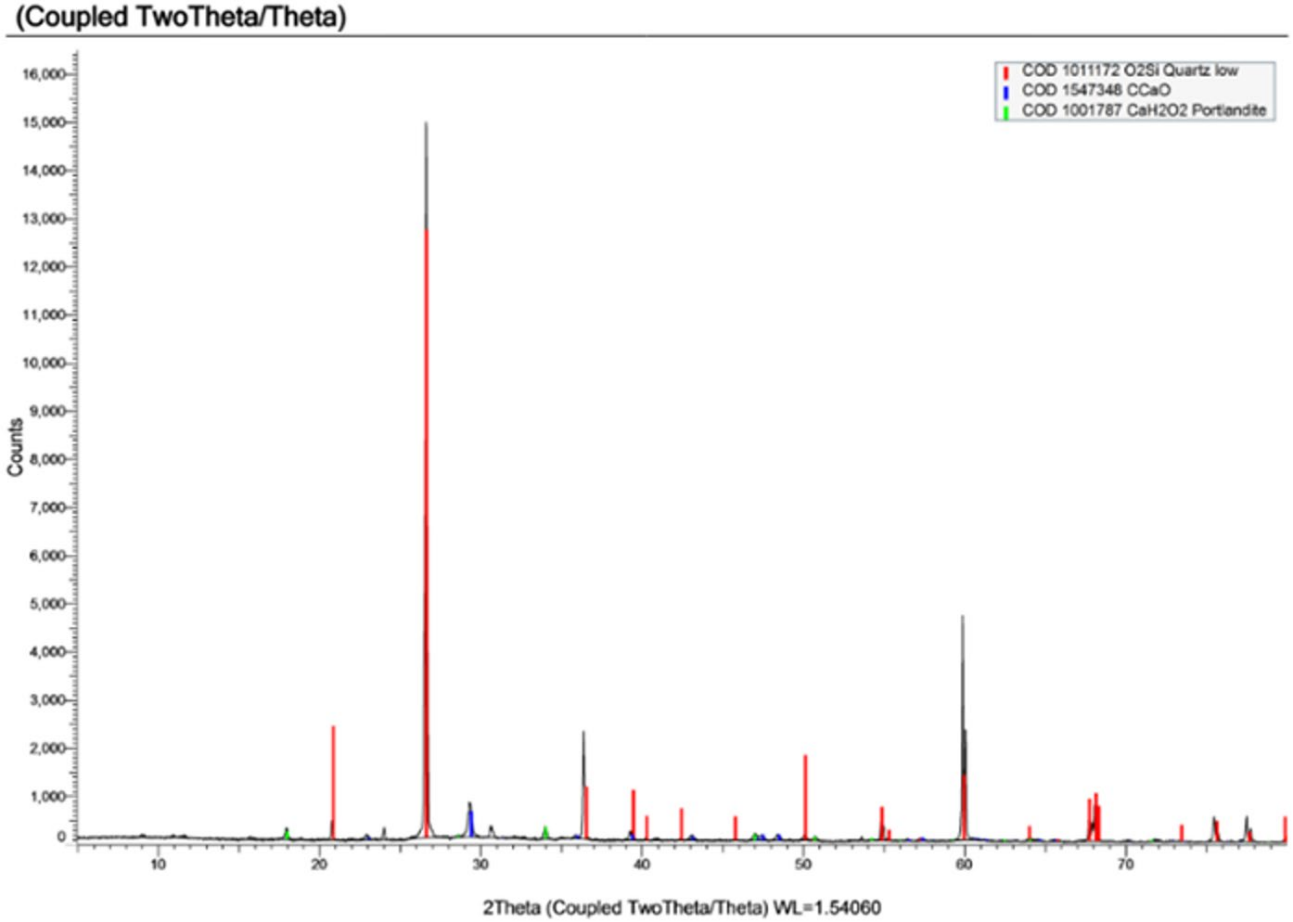
XRD of sample made with TETI.

Xray diffraction was performed on 3 samples extracted from specimens made using the 3 types of water under study and results are shown in Figs. 14, 15 and 16 and a conclusion of the important compounds are shown in Table 10.

X-ray diffraction (XRD) analysis revealed that the control mix (PW) exhibited strong crystalline phases associated with cement hydration, including gibbsite, corundum, and calcite. In contrast, both TEFI and TETI, mixed with treated industrial wastewater, showed diminished presence of these hydration products and instead higher proportions of inert or non-cementitious minerals such as quartz and K-feldspar. These results support the observed decrease in compressive strength and increase in setting time which is due to the decrease in the formation of hydration products.

**Table 10:** Major compounds result from XRD.

Type of Water used	Quartz (SiO_2_)	Calcite (CaCO_3_)	K-feldspar (AlKNaSi₃O₈)	Gibbsite (Al (OH)_3_)
PW	56.9%	19.6%	--	7.9%
TEFI	24.2%	2.3%	17.0%	--
TETI	85.0%	3.6%	--	--

### Phase III

This phase is focused on properties of cement paste and mortar, including initial and final setting time of cement paste and compressive strength of cement mortar.

#### Water consistency of cement paste

Water Consistency was carried out to determine the amount of water needed for normal consistency, which is the amount of water needed needed to achieve a drop of 10 mm of the standard cylindrical rod as per ASTM C 0187^27^. Amount of water needed for all types of water was 184 gm for 650 gm of cement. Therefore, water to cement ratio was 0.283.

#### Setting time of cement paste

**Fig. 17:**
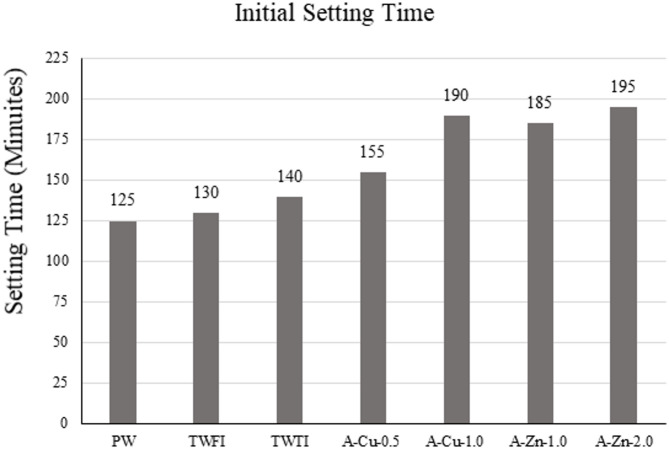
Initial setting time for different types of water used.

**Fig. 18:**
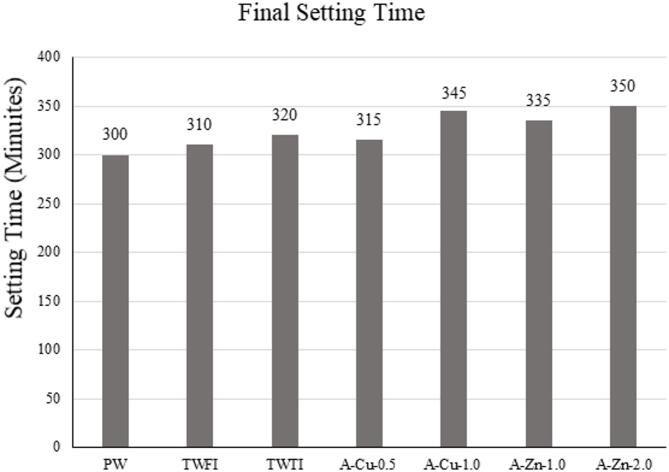
Final setting time for different types of water used.

Initial and final setting time tests according have been performed on the mixes and results are displayed in Figs. 17 and 18. The specimens made using treated wastewater shows clear retardation in the setting time. The increase in setting time in both TEFI and TETI is small and is probably due to the high COD & BOD numbers which indicates the presence of many impurities that affect the hydration reaction of cement and hence delay the setting time.

However, when looking at the artificial water results where the only impurities are heavy metals, it is noted that the increase in setting time is significant, and the value of retardation increases as the heavy metal concentration increases. This can be explained by the fact that heavy metal ions as Zn²⁺& Cu²⁺ interfere with the hydration of cement and are able to form insoluble hydroxides that precipitate on the surface of cement grains that slows down the hydration process.

By comparing the results to the code limits given in ASTM & BS, it is noted that the initial setting time retardation for TWFI, TWTI & A-Cu-0.5 are acceptable in comparison with limits. However, increasing the concentration of heavy metals higher than 0.5 gm/L leads to specimens being unacceptable in comparison with code limits. As for the final setting time, all 6 specimens are acceptable in comparison with code limits.

This retardation behavior aligns with the findings of^12,30,31^.

#### Compressive strength of cement mortar

7-day Compressive strength on standard mortar cubes has been tested according to ASTM C109^19^, and results are shown in Table 11. There is a general reduction in the compressive strength when replacing potable water with any type of TIWW. Using TEFI, TETI & A-Cu-0.5 leads to a reduction of 4.8%, 9.4% & 9.7% respectively, which is acceptable to all standards as previously mentioned. On the other hand, increasing heavy metal concentrations of more than 0.5 gm/L leads to a higher reduction exceeding 10% and hence these types of water are considered unacceptable to be used. This reduction can be explained by the fact that high amounts of oxygen demanding organic matter are found in both TEFI and TETI, this is based upon the observation that TETI has higher COD and BOD numbers compared to TEFI. For artificial water samples, increasing the percentages of heavy metals lead to decrease of compressive strength. This can also be linked to the retardation in hydration process that is accompanied by the increase in heavy metals percentages.

This reduction in compressive strength aligns with the findings of^9,10,12^.

**Table 11:** The 7-day compressive strength results of mortar cubes.

Type of water used	7-Day mortar compressive strength (MPa)	Reduction percentage (%)
PW	25.04	--
TEFI	23.82	4.8
TETI	22.69	9.4
A-Cu-0.5	22.61	9.7
A-Cu-1.0	21.46	14.3
A-Zn-1.0	21.73	13.2
A-Zn-2.0	17.87	28.6

### Phase IV results and discussion

This phase is focused on assessing any potential environmental hazard that could result from high concentration heavy metal water being used in cementitious products. This Hazard was evaluated by determining potential diffusion of heavy metals from a cementitious matrix (cement paste in this case) to a surrounding Leachant mass. This phase IV utilizes ASTM C1308^20^.

The Accelerated Leach Test was performed using three specimens prepared with different zinc concentrations: 0.5, 1.0, and 2.0 g/L. At the end of each specified time interval, leachate samples were collected and analyzed in the laboratory for zinc content. These intervals were selected to capture both short- and long-term leaching behavior.

Two parameters were calculated to characterize zinc leaching:

- *IFL (Incremental Fractional Leached)* the ratio of zinc released at each individual interval to the initial zinc content in the mortar cube.
- *CFL (Cumulative Fractional Leached)* the ratio of the total zinc released from the beginning of the test up to a given interval to the initial zinc content in the mortar cube.

Tables 12, 13 and 14 show both IFL and CFL results for 3 samples prepared.

**Table 12:** Leaching results for specimen no.1 (2.0 g/L).

Time interval (days)	Quantity of Zn measured (mg)	IFL (mg/mg)	CFL (mg/mg)
0.0833	0.42	0.01085	0.01085
0.20833	0.14	0.00362	0.01446
0.70833	0.12	0.00301	0.01756
1	0.09	0.00232	0.01988
2	0.08	0.00206	0.02195
4	0.04	0.00103	0.02505
6	0.0	0.0	0.02531
8	0.0	0.0	0.02531
10	0.0	0.0	0.02531

**Table 13:** Leaching results for specimen no.2 (1.0 g/L).

Time interval (days)	Quantity of Zn measured (mg)	IFL (mg/mg)	CFL (mg/mg)
0.0833	0.38	0.01963	0.01963
0.20833	0.13	0.00671	0.02634
0.70833	0.12	0.00620	0.03254
1	0.10	0.00517	0.03770
2	0.08	0.00413	0.04184
4	0.04	0.00258	0.04648
6	0.0	0.0	0.04700
8	0.0	0.0	0.04700
10	0.0	0.0	0.04700

**Table 14:** Leaching results for specimen no.3 (0.5 g/L).

Time Interval (days)	Quantity of Zn measured (mg)	IFL (mg/mg)	CFL (mg/mg)
0.0833	0.29	0.02996	0.02996
0.20833	0.09	0.00929	0.03926
0.70833	0.07	0.00723	0.04649
1	0.04	0.00413	0.05062
2	0.02	0.00206	0.05269
4	0.0	0.0	0.05269
6	0.0	0.0	0.05269
8	0.0	0.0	0.05269
10	0.0	0.0	0.05269

The final cumulative fractional leached (CFL) values remained below 0.06 for all specimens, indicating that more than 94% of the initial zinc content remained immobilized within the cementitious matrix throughout the testing period. In practical terms, a cumulative release of less than 5–6% under accelerated conditions demonstrate strong chemical stabilization and physical bounding of heavy metals.

## Summary and conclusions

This study evaluated the feasibility of using treated industrial wastewater from textile and food industries—as well as synthetic wastewater containing controlled heavy metal concentrations—as a substitute for potable water in concrete and mortar production. Through chemical characterization, mechanical and durability testing, microstructural analysis, and leaching assessments, the research compared performance outcomes against code requirements to determine both technical suitability and environmental safety.

In addition to the above-mentioned results, the experimental program effectively was able to save from 70 to 100 L of fresh water during the preparation of the concrete/mortar samples used in the experimental phases, by replacing the fresh potable water by the treated industrial wastewater.

Overall, the results show that properly monitored treated wastewater can be used in cementitious materials without significant reductions in performance or increased environmental risk. The specific conclusions are as follows:

1. Tρeατeδ ωαστeωατeρ quαliτy vαρieσ βy iνδuστρy, βuτ αll τeστeδ σαµπleσ µeτ coδe liµiτσ δeσπiτe higheρ COΔ, BOΔ, ανδ heαvy µeταl πeρceνταgeσ, eσπeciαlly iν τexτile ωαστeωατeρ.
2. Using treated wastewater caused small compressive strength reductions in concrete (< 10%) due to mild hydration retardation and disruption of the interfacial transition zone caused by organic matter and dissolved impurities.
3. Tensile and flexural strengths of concrete were minimally affected, with reductions under 5%.
4. Concrete exhibited reduced Sorpitivity compared to the control mix, due to pore-blocking effects of organic compounds. In contrast, rapid chloride penetration values increased, reflecting higher ionic conductivity rather than increased pore connectivity.
5. Cement paste and mortar tests revealed that heavy metal concentrations exceeding 0.5 g/L cause unacceptable setting time delays and compressive strength losses exceeding 10%.
6. Accelerated leaching tests demonstrated minimal release of zinc from cementitious matrices, indicating effective immobilization of heavy metals and low environmental risk.

Overall, treated industrial wastewater can be safely utilized as mixing water in concrete and mortar for non-critical and structural applications, provided that chemical constituents are routinely monitored and maintained within acceptable limits.

## Recommendations for future research

Based on the outcomes of this study, the following recommendations are proposed for future research:

1. *Expansion to diverse industrial wastewater sources* Future studies should investigate treated wastewater from a wider range of industrial sectors to capture variability in chemical composition, organic content, and heavy metal concentrations, and to support the development of generalized acceptance criteria for wastewater reuse in concrete.
2. *Long-term durability and exposure performance* Extended investigations are recommended to assess the long-term durability of concrete produced with treated industrial wastewater under aggressive environmental conditions, including chloride exposure, carbonation, sulfate attack, and freeze–thaw cycles.
3. *Alternative applications and curing strategies* The use of treated industrial wastewater as curing water, as well as its application in cement masonry units, precast elements, and non-structural concrete products, should be explored as practical and low-risk pathways for large-scale implementation.
4. *Microstructural analysis and sustainability assessment* Future work should integrate advanced microstructural characterization techniques and life cycle assessment to better understand transport mechanisms, hydration behavior, and the overall environmental benefits of replacing potable water with treated industrial wastewater.

## Data Availability

The data will be made available by the authors upon reasonable request. Please contact the corresponding author, Dr. Ayman Shamseldein, for access.
